# Construction and Comprehensive Analyses of a Competing Endogenous RNA Network in Tumor-Node-Metastasis Stage I Hepatocellular Carcinoma

**DOI:** 10.1155/2020/5831064

**Published:** 2020-02-11

**Authors:** Xuefeng Gu, Hongbo Li, Ling Sha, Wei Zhao

**Affiliations:** ^1^Department of Liver Disease, The Second Hospital of Nanjing, Medical School, Southeast University, Nanjing 210003, China; ^2^Department of Hepatology, Infectious Diseases Hospital Affiliated with Soochow University, Suzhou 215000, China; ^3^Department of Neurology, Affiliated Drum Tower Hospital of Nanjing University Medical School, Nanjing 210008, China

## Abstract

**Background:**

Long noncoding RNAs (lncRNAs) can function as competing endogenous RNAs (ceRNAs) and interact with microRNAs (miRNAs) to regulate target gene expression, which can greatly influence tumor development and progression. Different tumor-node-metastasis (TNM) stages of hepatocellular carcinoma (HCC) defined by the American Joint Committee on Cancer (AJCC) have different clinical results. Our purpose was to comprehensively analyze differentially expressed (DE) lncRNAs, miRNAs, and mRNAs in stage I HCC and identify prognosis-associated RNAs.

**Methods:**

RNA-seq data were obtained from The Cancer Genome Atlas (TCGA) database. A stage I HCC-associated miRNA-lncRNA-mRNA network was constructed. Next, Gene Ontology (GO) and Kyoto Encyclopedia of Genes and Genomes (KEGG) enrichment pathway analyses of ceRNA-associated DEmRNAs were performed using Database for Annotation, Visualization, and Integrated Discovery (DAVID) 6.8 and Clusterprofile in the R package. The protein-protein interaction (PPI) network of the above mRNAs was then constructed using STRING. Finally, the association between lncRNAs and mRNAs in the ceRNA network and prognosis of patients was further analyzed. Linear regression analysis of the above lncRNAs and mRNAs associated with overall survival was performed.

**Results:**

After a comparison between HCC and adjacent nontumor tissues, 778 lncRNAs, 1608 mRNAs, and 102 miRNAs that were abnormally expressed were identified. The ceRNA network was composed of 56 DElncRNAs, 14 DEmiRNAs, and 30 DEmRNAs. Functional analysis results showed that 30 DEmRNAs were enriched in 14 GO biological process categories and 6 KEGG categories (false discovery rate (FDR) < 0.05). A PPI network was composed of 22 nodes and 58 edges. We detected 4 DElncRNAs (BPESC1, AC061975.6, AC079341.1, and CLLU1) and 6 DEmRNAs (CEP55, E2F1, E2F7, EZH2, G6PD, and SLC7A11) that had significant influences on the overall survival (OS) of stage I HCC patients (*P* < 0.05). lncRNA BPESC1 was positively correlated with mRNA CEP55 via miR-424, and lncRNA AC061975.6 was positively correlated with mRNA E2F1 via miR-519d.

**Conclusion:**

Our study identified novel lncRNAs and mRNAs that were associated with the progression and prognosis of stage I HCC and further investigated the regulatory mechanism of lncRNA-mediated ceRNAs in the development of stage I HCC.

## 1. Introduction

Hepatocellular carcinoma (HCC) is the 6^th^ most common cancer in the world and is the 4^th^ leading cause of cancer-related death [1]. Cirrhosis, chronic hepatitis B and C virus infection, dietary aflatoxin exposure, and alcoholism are the most common risk factors for HCC [2, 3]. Although the diagnostic methods and surgical treatment measures for HCC continue to improve, the 5-year survival rate of late-stage HCC patients is still very low [4]. Many clinical treatments for tumors are performed based on the tumor-node-metastasis (TNM) staging system of the American Joint Committee on Cancer (AJCC) [5]. Early AJCC stage I HCC patients are most likely to be cured through treatment methods that include radiofrequency ablation (RFA), surgical resection, or liver transplantation [6]. Stage I HCC patients with 1 single tumor that has no vascular invasion have higher survival rates than do HCC patients in other stages [7]. However, the treatment effect on early HCC is still not high, and many patients eventually die from tumor recurrence and liver failure [8]. The 5-year survival rate of stage I HCC is still only approximately 50% and is greatly lower than that of stage I cancers in other organs [9]. The molecular mechanism for mediating the recurrence of early-stage HCC is still not clear. Therefore, identifying biomarkers that can accurately and reliably predict HCC prognosis in the early stage of HCC is urgently needed in order to understand the molecular mechanism underlying the poor survival period of early-stage HCC.

The relationship among long noncoding RNAs (lncRNAs), microRNAs (miRNAs), and messenger RNAs (mRNAs) is complicated. In 2011, Salmena et al. [10] proposed the hypothesis of competing endogenous RNAs (ceRNAs). The ceRNA hypothesis considers that lncRNAs not only can directly participate in the regulation of target gene expression but also may contain some core seed sequences that can adsorb corresponding miRNAs to further interfere with target genes mRNA abundance by influencing the number of miRNAs, thus affecting gene expression. Martini et al. [11] showed that the expression levels of lnc-SERTAD2-3, lnc-SOX4-1, lncHRCT1-1, and PVT1 in stage I epithelial ovarian cancer (EOC) were independent prognostic markers associated with recurrence and poor prognosis. Based on an analysis of The Cancer Genome Atlas (TCGA) database, RNA sequencing, and Reverse Transcription-Polymerase Chain Reaction (RT-PCR) experiments, Tian et al. [12] showed that 8 out of 12 candidate lncRNAs (LINC00963, NR2F2-AS1, LINC00515, LINC00162, LINC00312, MGC27382, LINC00472, and FENDRR) were significantly upregulated in stage I lung adenocarcinoma (LUAD) tissues. Ma et al. [13] showed that the lncRNA PAPAS could promote HCC through an interaction with miR-188-5p. The plasma level of PAPAS could effectively differentiate between stage I HCC patients and healthy controls. However, no study has targeted the regulatory mechanism underlying lncRNA-mediated ceRNAs in early-stage (stage I) HCC and the diagnostic and prognostic values of genes in the ceRNA network that participate in stage I HCC.

Recent studies have shown that Bai et al. constructed an HCC-associated deregulated ceRNA network consisting of 37 lncRNAs, 10 miRNAs, and 26 mRNAs after excluding lncRNAs that were localized only in the nucleus [14]. In our study, we investigated the RNA expression conditions in 171 cases (172 cases of miRNAs) of stage I HCC and 50 cases of nontumor normal liver tissues based on TCGA data. We constructed a ceRNA network containing 56 lncRNAs, 14 miRNAs, and 30 mRNAs in stage I HCC. Next, Gene Ontology (GO) and Kyoto Encyclopedia of Genes and Genomes (KEGG) functional enrichment and protein-protein interaction (PPI) analyses were performed to elucidate the underlying mechanism. Finally, the prognostic values of the above lncRNAs, miRNAs, and mRNAs involved in the ceRNA network for determining the overall survival (OS) of stage I HCC patients were analyzed. We believe that our study might provide novel prognostic biomarkers for survival prediction and targeted treatment of early-stage HCC.

## 2. Materials and Methods

### 2.1. Data Acquisition and Selection

The gdc-client tool was used to download raw sequencing data of HCC-associated mRNA and miRNA expression from the TCGA database (https://portal.gdc.cancer.gov/). Complete clinical data of the corresponding patients were further downloaded from the cBioPortal (http://www.cbioportal.org/) website. Liver tissue specimens of stage I HCC patients (171 cases for mRNAs and lncRNAs and 172 cases for miRNAs) and 50 specimens from normal individuals were selected. The corresponding mRNA-seq, lncRNA-seq, and miRNA-seq data were further obtained. We then used the R package of edgeR to homogenize the TCGA raw data using the trimmed mean of *M*-values (TMM) method. If there were multiple data points for RNA expression, the average expression value was regarded as the expression value of the corresponding gene. Finally, the RNA expression level was converted to a log2 value. Our study entirely followed the publication guidelines of TCGA (https://cancergenome.nih.gov/publications/publicationguidelines); therefore, approval from an ethics committee was not required.

### 2.2. Identification of Differentially Expressed (DE) RNAs

Based on the annotation of the Ensembl database (http://www.ensembl.org/index.html), DElncRNAs and DEmRNAs were defined and encoded. mRNAs, lncRNAs, and miRNAs differentially expressed in stage I HCC and normal liver tissues were screened using the edgeR R package (version: 3.22.5) in R software (version 3.5.2). Statistical significance was defined as log2 fold change > 2 and a *P* value <0.01. Volcano plots and heatmaps of DERNAs were plotted using the ggplots and heatmap packages.

### 2.3. Construction of the lncRNA-miRNA-mRNA ceRNA Network

Using the miRcode database (http://www.mircode.org/), miRNAs that interacted with DElncRNAs were searched. The obtained miRNAs were modified using the StarBase database (http://starbase.sysu.edu.cn/). To further study lncRNA functions, the Perl program (version: 5.26.1) was used to predict miRNA target genes using 3 databases: miRDB (http://www.mirdb.org/), TargetScan (http://www.targetscan.org/), and miRTarBase (http://mirtarbase.mbc.nctu.edu.tw/php/index.php). The intersecting mRNAs obtained using the 3 prediction methods were retained. The intersection between these mRNAs and DEmRNAs was then obtained using the VennDiagram R package (version: 1.6.20). DEmRNAs that were involved in the construction of the ceRNA network were obtained. The lncRNA-miRNA-mRNA ceRNA network based on the “ceRNA hypothesis” was constructed and visualized using Cytoscape v3.7.1.

### 2.4. Functional Enrichment Analysis

To understand the underlying biological mechanism of DEmRNAs in the ceRNA crosstalk network, the Database for Annotation, Visualization, and Integrated Discovery (DAVID) bioinformatics database (https://david-d.ncifcrf.gov/) was used to execute GO biological enrichment analyses. The ClusterProfiler v3.12.0 package of R [15] was used to analyze KEGG pathways. The GOplot package of R was used to display the results of the GO and KEGG analyses. False discovery rate (FDR) < 0.05 was set as the cut-off standard.

### 2.5. Construction of the PPI Network

The STRING database (version 11.0, https://string-db.org/) is a public data source that can provide information regarding the interaction between known proteins and predicted proteins. To elucidate the potential protein-protein relationship between DEmRNAs involved in the ceRNA network, a PPI network was constructed using STRING. Interactions with confidence scores above 0.4 were considered significant and were retained. Genes with a number of connections ≥5 were considered hub genes in the PPI network.

### 2.6. Survival Analysis

The association between RNAs and survival time was analyzed using Kaplan–Meier (KM) curves and the log-rank test. The survival package and ggplot2 package in R language were used for statistical analyses and plotting. *P* < 0.05 was set as the cut-off value.

## 3. Results

### 3.1. Differentially Expressed mRNAs, lncRNAs, and miRNAs

**Figure 1:**
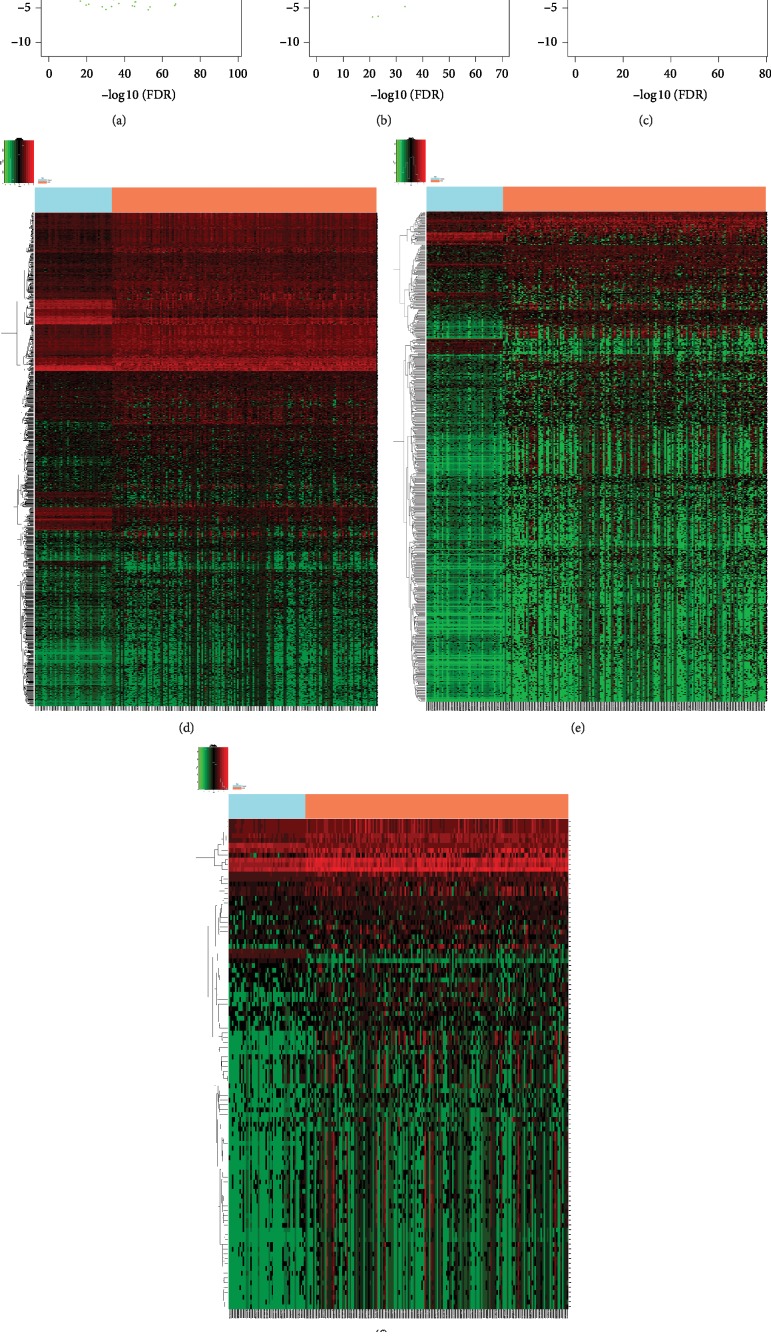
Specific lncRNA-related ceRNA network and characteristics of the constituent lncRNAs in stage I HCC patients. The volcano plot shows the expression profiles of mRNAs (a), lncRNAs (b), and miRNAs (c). Red dots indicate upregulated RNAs, and green dots indicate downregulated RNAs. Heatmaps of differentially expressed RNAs in different samples: (d) mRNAs, (e) lncRNAs, and (f) miRNAs. The *y*-axis represents RNAs and the *x*-axis represents patient samples; red denotes upregulation and green denotes downregulation. ceRNA: competing endogenous RNA; HCC: hepatocellular carcinoma; lncRNA: long noncoding RNA; miRNA: microRNA.

Detailed clinical information of the included patients is shown in Table 1. Differentially expressed (DE) mRNAs and DElncRNAs were identified when comparing 171 stage I HCC tissues and 50 normal liver tissues. A total of 1608 DEmRNAs were identified, of which 1385 mRNAs were upregulated and 223 mRNAs were downregulated. In addition, a total of 778 DElncRNAs were identified, of which 722 lncRNAs were upregulated and 56 lncRNAs were downregulated. Furthermore, a total of 102 DEmiRNAs (98 upregulated genes and 4 downregulated genes) were identified when comparing 172 stage I hepatocellular carcinoma (HCC) tissues and 50 normal liver tissues. The volcano plots of the mRNAs, lncRNAs, and miRNAs are shown in Figures 1(a)–1(c), respectively; their heatmaps are shown in Figures 1(d)–1(f), respectively.

### 3.2. Construction of the Competing Endogenous RNAs (ceRNAs) Network for Stage I HCC

**Figure 2:**
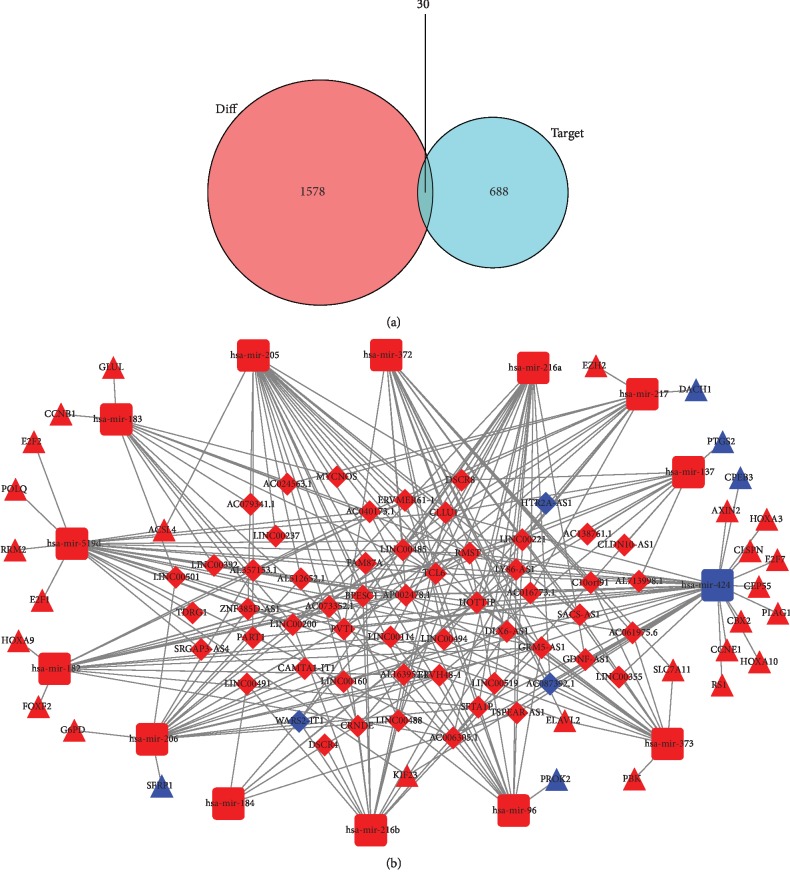
The ceRNAs network in stage I HCC patients. (a) Venn diagram of DEmRNAs involved in the ceRNA network. (b) The ceRNA network of lncRNAs-miRNAs-mRNAs involved in HCC. Diamonds represent IncRNAs, round rectangles represent miRNAs, and triangles represent mRNAs. The nodes highlighted in red and blue indicate up- and downregulation, respectively. DEmRNA: differentially expressed mRNA.

To further investigate how lncRNAs mediated mRNAs regulation through interactions with miRNAs in stage I HCC, a lncRNA-miRNA-mRNA (ceRNA) network based on the above data was constructed and visualized using Cytoscape v3.7.1. First, we used the alignment file of the miRcode database to align DElncRNAs and miRNAs. Next, we extracted the pairs of miRNAs that interact with DElncRNAs to obtain the DElncRNA alignment file in the miRcode database. Finally, we aligned the obtained alignment results with DEmiRNAs and obtained a total of 212 pairs with successful alignment records; that is, 212 interacting lncRNA and miRNA pairs were identified from DElncRNAs and DEmiRNAs. Among these 212 interaction pairs, there were 56 lncRNAs and 14 miRNAs. We then searched for the target mRNAs of these 14 miRNAs using the miRTarBase, miRDB, and TargetScan databases and obtained a total of 718 target mRNAs. We intersected these 718 genes with the original 1608 DEmRNAs and obtained 30 DEmRNAs as nodes of the ceRNA network (Figure 2(a)). Combining the miRNA-mRNA interactions and lncRNA-miRNA interactions, a lncRNA-miRNA-mRNA network was constructed. This network was composed of 56 DElncRNAs, 14 DEmiRNAs, and 30 DEmRNAs (Figure 2(b)).

### 3.3. Functional Analysis of ceRNA Network-Associated DEmRNAs

**Figure 3:**
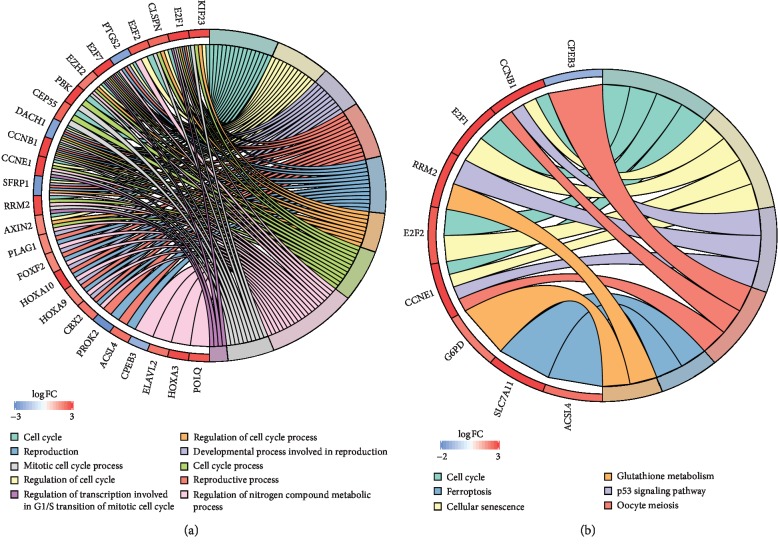
Enrichment analysis of DEmRNAs involved in the ceRNA network. (a) The top 10 significantly enriched pathways identified in the DEmRNA GO enrichment analysis. (b) Significantly enriched KEGG pathways of DEmRNAs (FDR < 0.05). GO: Gene Ontology; KEGG: Kyoto Encyclopedia of Genes and Genomes; FDR: false discovery rate.

Functional analysis results showed that 30 DEmRNAs in the above ceRNA network were enriched in 14 Gene Ontology (GO) biological process categories and 6 Kyoto Encyclopedia of Genes and Genomes (KEGG) categories (false discovery rate (FDR) < 0.05). The results are shown in Tables 2 and 3. The top 10 GO terms were cell cycle, regulation of cell cycle, the developmental process involved in reproduction, reproductive process, reproduction, regulation of cell cycle process, cell cycle process, regulation of nitrogen compound metabolic process, mitotic cell cycle process, and transcription factor complex (Figure 3(a)).  Figure 3(b) shows the significantly enriched pathways for these DEmRNAs after KEGG pathway enrichment analyses: cell cycle, cellular senescence, p53 signaling pathway, oocyte meiosis, ferroptosis, and glutathione metabolism.

### 3.4. Protein-Protein Interaction (PPI) Network of ceRNA Network-Associated DEmRNAs

**Figure 4:**
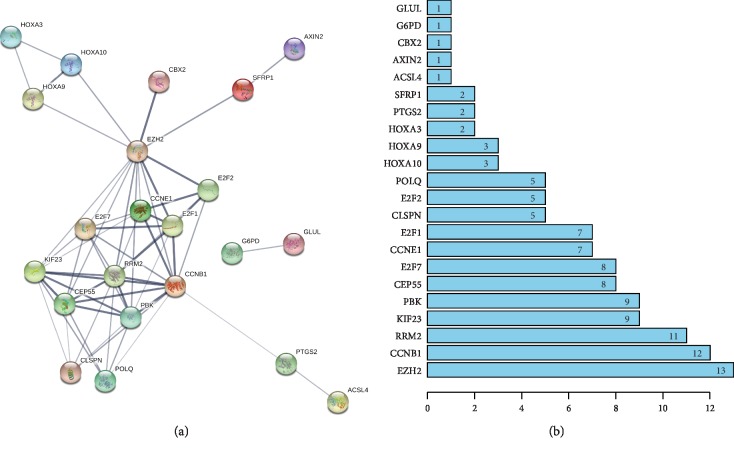
Identification of hub genes from the PPI network. (a) The PPI network of ceRNA network-associated DEmRNAs. (b) Information about nodes in the PPI network. PPI: protein-protein interaction.

A PPI network was constructed using STRING and was composed of 22 nodes and 58 edges (Figure 4(a)). According to this network, there were a total of 12 hub genes (degree ≥ 5). These major hub genes included EZH2, CCNB1, RRM2, KIF23, PBK, CEP55, E2F7, CCNE1, E2F1, CLSPN, E2F2, and POLQ (Figure 4(b)). The gene with the highest degree (degree = 13) was enhancer of zeste homolog 2 (EZH2) (Figure 4(b)).

### 3.5. Overall Survival-(OS-) Associated lncRNAs and mRNAs in the ceRNA Network

**Figure 5:**
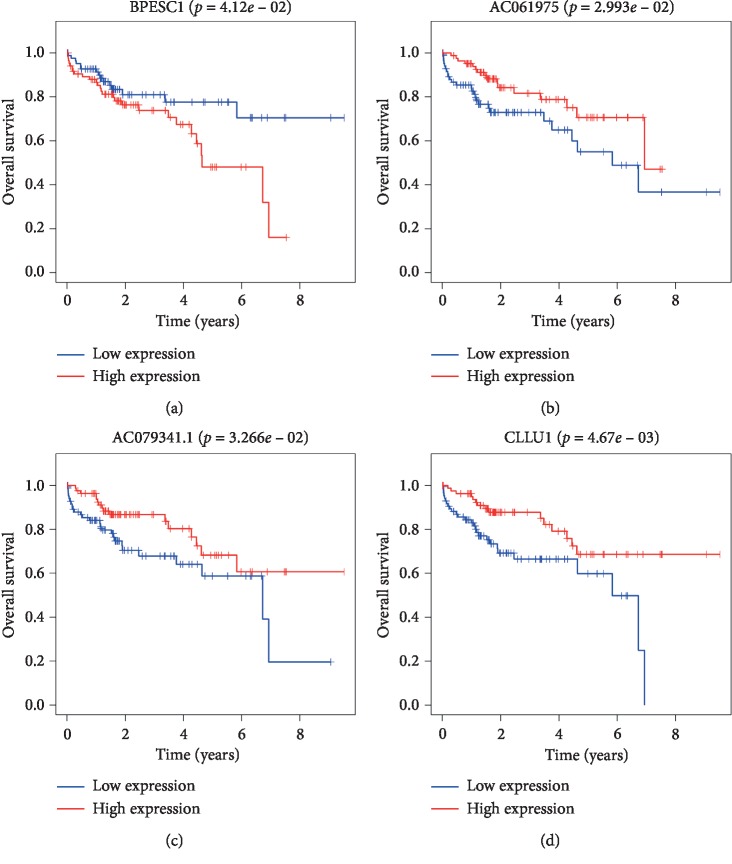
Kaplan–Meier survival curves of 4 DElncRNAs involved in the ceRNA network associated with overall survival in patients with stage I HCC (*P* < 0.05). DElncRNA: differentially expressed lncRNA.

**Figure 6:**
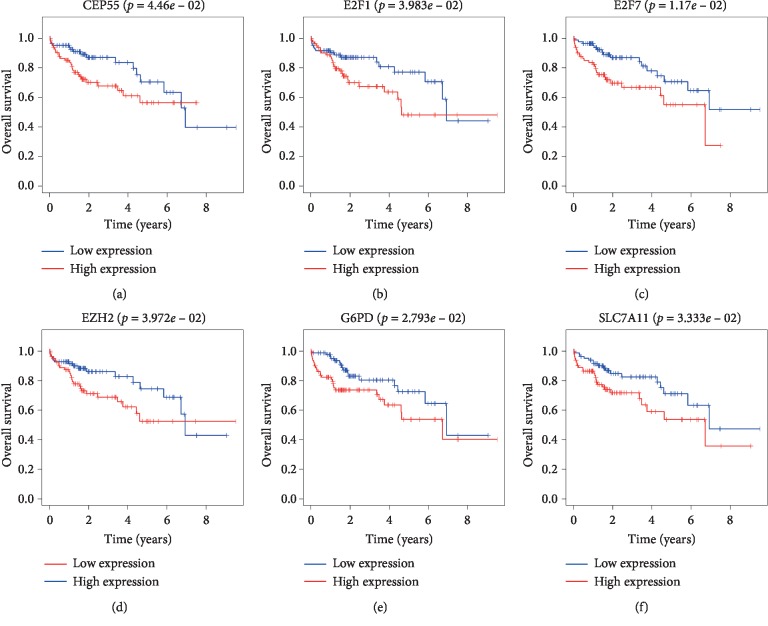
Kaplan–Meier survival curves of 6 DEmRNAs involved in the ceRNA network associated with overall survival in patients with stage I HCC (*P* < 0.05).

**Figure 7:**
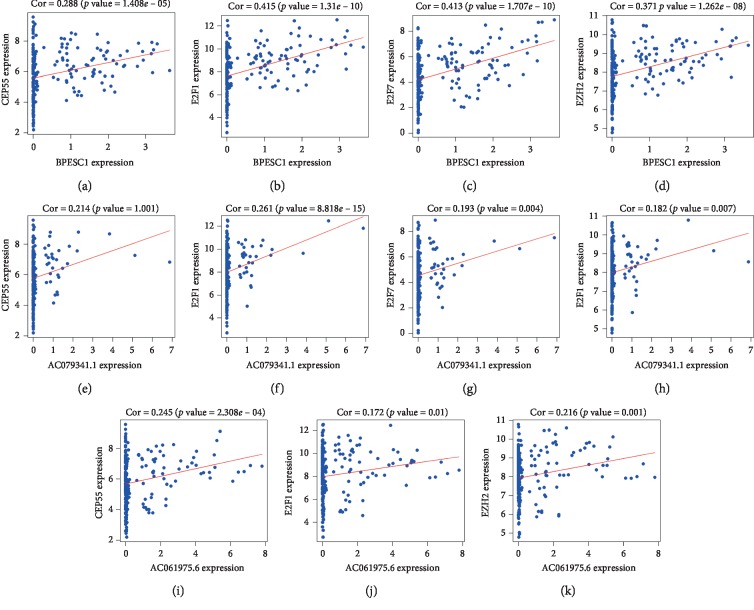
Correlation analysis of DEGs linear regression analysis between lncRNAs and mRNAs associated with overall survival. Linear regression of BPESC1 (a–d), AC079341.1 (e–h), and AC061975.6 (i–k) versus related DEmRNA expression level (*P* < 0.05). The red line represents the linear model fitted by the dots in each figure. DEGs: differentially expressed genes.

**Figure 8:**
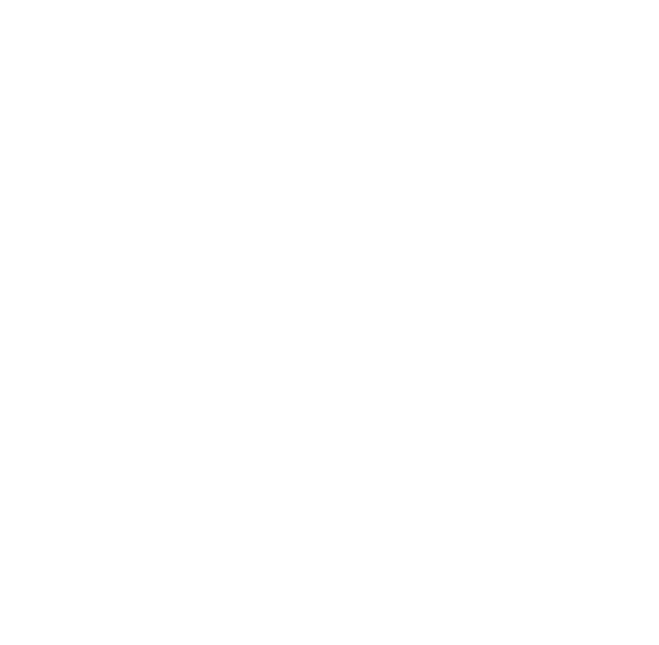
Flow chart of the bioinformatics analysis.

To elucidate the association between DElncRNAs in ceRNAs and the prognosis of HCC patients, KM curve analyses were performed. The results showed that 4 out of 56 DElncRNAs were significantly associated with OS (*P* < 0.05). BPESC1 negatively correlated with patient OS, whereas highly expressed AC061975.6, AC079341.1, and CLLU1 significantly prolonged the survival time of patients with stage I HCC (Figures 5(a)–5(d)). Next, we performed KM curve analyses on 30 DEmRNAs in ceRNAs to study the association between DEmRNAs and the OS of patients with stage I HCC. The results suggested that 6 DEmRNAs were significantly associated with OS. These 6 DEmRNAs (CEP55, E2F1, E2F7, EZH2, G6PD, and SLC7A11) negatively correlated with OS (Figures 6(a)–6(e)). No significant differences were observed between DEmiRNAs and the OS of patients with stage I HCC. Details of DEmRNAs and DElncRNAs associated with overall survival are shown in Table 4. Linear regression analysis of the 4 lncRNAs and 6 mRNAs associated with overall survival was performed. 11 lncRNA-mRNA pairs are positively correlated (Figures 7(a)–7(k), *P* < 0.05). Next, we explored whether shared miRNAs existed between the lncRNAs and mRNAs. We found that lncRNA BPESC1 was positively correlated with mRNA CEP55 through miRNA miR-424, and lncRNA AC061975.6 was positively correlated with mRNA E2F1 through miRNA miR-519d.  Figure 8 shows a flow diagram of the bioinformatics analysis.

## 4. Discussion

During disease diagnosis, the progression level of cancer is the key factor defining treatment methods and evaluating successful treatment results. Therefore, the elucidation of molecular mechanisms in different progression stages of HCC is urgently needed. In recent years, with the advance of studies on the complicated interaction among different RNA types and RNA crosstalk in the gene regulation network, miRNAs and their ceRNA targets have been confirmed; for example, lncRNAs and mRNAs can form a complicated ceRNA network [16]. Different studies have constructed ceRNA networks with DERNAs in HCC and normal tissues based on next-generation sequencing or microarray data [17–19]. However, comprehensive analysis of ceRNA networks based on differential expression profile data for stage I HCC is lacking. Discovery and diagnosis of early-stage HCC are very critical for HCC treatment; therefore, identifying novel therapeutic targets and prognostic markers in stage I is particularly important.

In this study, we identified 778 lncRNAs, 1608 mRNAs, and 102 miRNAs that were abnormally expressed in HCC tissue based on RNA-seq data in the TCGA database. We then constructed a stage I HCC-associated miRNA-lncRNA-mRNA network. The results indicated that crosstalk among these RNAs might be an important feature of stage I HCC. Notably, among the 6 mRNAs identified in the miRNA-lncRNA-mRNA network that were associated with prognosis, E2F1 and E2F7 both belong to the E2F transcription factor family; the E2F family plays an important role in the regulation of cell cycle [20]. The results of our GO and KEGG analyses also suggested that the 6 mRNAs were mainly enriched in cell cycle pathways, including the cell cycle, regulation of the cell cycle, and cell cycle processes. CEP55, EZH2, and SLCA11 are highly expressed in HCC and play critical regulatory roles in cancers [21–23]. They were key nodes that made up the stage I HCC ceRNA network in our study and were key potential prognostic genes in stage I HCC, results that are consistent with a Gene Expression Omnibus (GEO) database study by Yue et al. [24]. It is notable that the above genes exhibited high degrees in the PPI network constructed using the ceRNA network-associated DEmRNAs and were major hub genes. These results suggested that they played very important roles in the biological processes of stage I HCC.

Abnormal lncRNA expression is involved in various human tumors including HCC. In recent years, intensive studies have been carried out on lncRNAs. The involvement of lncRNAs in the mechanisms of many cancers has been explored, and the same lncRNA can regulate many types of cancers at the same time. In our study on ceRNA network-associated lncRNAs, AC061975.6, AC079341.1, BPESC1, and CLLU1 were associated with the OS of stage I HCC patients. Buhl et al. [25] showed that CLLU1 was significantly upregulated in CLL cells and that the OS of CLL patients was associated with the CLLU1 expression level. When the expression level of CLLU1 increased 1-fold, the risk of early death increased by 7%. CLLU1 expression had a stronger prognostic meaning in patients younger than 70 years of age, whereas it did not have prognostic meaning in patients 70 years of age or older [26]. Cahill et al. [27] showed that high levels of DNA methylation in the CLLU1 gene were present in immunoglobulin heavy chain- (IGHV-) mutated (IGHV-M) CLL and in normal B cells of IGHV-unmutated (IGHV-UM) patients. High CLLU1 expression and the presence of the IGHV-UM gene were associated with poor clinical results [25, 28]. Yue et al. [24] used the GEO database to construct a ceRNA regulatory network of HCC. CLLU1 was a key candidate lncRNA. It was upregulated in HCC tissues and was significantly associated with the prognosis of HCC patients.

Wang et al. [29] collected microarray data sets of 26 triple-negative breast cancer (TNBC) patients who received neoadjuvant chemotherapy (NAC). Receiver operating characteristic (ROC) analyses were performed on DElncRNAs to evaluate their predictive value in pathological complete response to NAC. The results suggested that BPESC1 was a marker with better prediction value. However, the function of BPESC1 has not been studied in HCC and other tumors, and thus, an investigation should be performed. In addition, there is little known about the other 2 potential prognostic lncRNAs (AC061975.6 and AC079341.1). Therefore, the functions of these potential prognostic lncRNAs in HCC and other cancers should be further studied and elucidated. Although there is currently no direct evidence of their involvement in tumor development, we found that BPESC1, AC061975.6, and AC079341.1 were associated with the prognosis of patients with stage I HCC.

In addition, Bai et al. [14] used the TCGA database to construct a ceRNA regulatory network of HCC in a recent study. The KM curve analysis showed that 15 lncRNAs, 3 miRNAs, and 16 mRNAs at the nodes of the constructed ceRNA network for HCC were significantly related to the overall survival rate for HCC patients. In particular, for these overall survival-related RNAs, high expression levels of the lncRNA BPESC1 and the 5 mRNAs CEP55, E2F1, EZH2, E2F7, and SLC7A11 were related to short survival time, and the expression level of CLLU1 was positively correlated with overall survival. These findings are consistent with our conclusions. Our results indicated that the lncRNAs AC061975.6 and AC079341.1 and the mRNA G6PD were related to the overall survival, whereas these RNAs were not detected by Bai et al., this discrepancy may be mainly attributable to the construction of different ceRNA networks for HCC with different clinical characteristics.

## 5. Conclusions

In summary, through the analyses of related stage I HCC data obtained from the TCGA database, the expression profiles of lncRNAs, miRNAs, and mRNAs were obtained. A lncRNA-miRNA-mRNA ceRNA network was successfully constructed. Six DEmRNAs and 4 DElncRNAs involved in the ceRNA network that had prognostic value were identified. BPESC1 was positively correlated with mRNA CEP55 via miR-424, and AC061975.6 was positively correlated with mRNA E2F1 via miR-519d. Some of these have been reported as promising biomarkers for diagnosis and prognosis.

## Figures and Tables

**Table 1:** Clinical characteristics of the included patients.

Characteristics	Number of sample size, *n* = 171 (%) (mRNA, lncRNA)	Number of sample size, *n* = 172 (%) (miRNA)
Age (years)		
≥60	95 (55.56)	95 (55.23)
<60	76 (44.44)	77 (44.77)

Gender		
Female	50 (29.24)	49 (28.49)
Male	121 (70.76)	123 (71.51)

Family history		
Yes	52 (30.41)	51 (29.65)
No	119 (69.59)	121 (70.35)

Race		
White	78 (45.61)	77 (44.77)
Asian	81 (47.37)	81 (47.09)
Black or african american	8 (4.68)	8 (4.65)
NA	4 (2.34)	6 (3.49)

Tumor status		
With tumor	44 (25.73)	46 (26.74)
Tumor free	117 (68.42)	116 (67.44)
NA	10 (5.85)	10 (5.81)

Hepatitis B virus infection		
Yes	69 (40.35)	70 (40.70)
No	96 (56.14)	96 (55.81)
NA	6 (3.51)	6 (3.49)

Histologic grade		
Grade 1	25 (14.62)	25 (14.53)
Grade 2	80 (46.78)	80 (46.51)
Grade 3	55 (32.16)	56 (32.56)
Grade 4	10 (5.85)	11 (6.40)
NA	1 (0.58)	0 (0.00)

Vascular invasion		
Macro	5 (2.92)	5 (2.91)
Micro	17 (9.94)	18 (10.47)
None	137 (80.12)	138 (80.23)
NA	12 (7.02)	11 (6.40)

Residual tumor		
R0	154 (90.06)	155 (90.12)
R1	5 (2.92)	5 (2.91)
R2	0 (0.00)	0 (0.00)
RX	9 (5.26)	9 (5.23)
NA	3 (1.75)	3 (1.74)

Vital status		
Living	129 (75.44)	131 (76.16)
Deceased	42 (24.56)	41 (23.84)

Relapse		
Yes	62 (36.26)	65 (37.79)
No	88 (51.46)	88 (51.16)
NA	21 (12.28)	19 (11.05)

**Table 2:** The top 10 GO terms enrichment for DEmRNAs involved in the ceRNA network using the DAVID database.

GO ID	Term	Genes	Gene count	FDR
GO: 0007049	Cell cycle	KIF23, E2F1, CLSPN, E2F2, PTGS2, E2F7, EZH2, PBK, CEP55, DACH1, CCNB1, CCNE1, SFRP1, RRM2, AXIN2	15	0.000

GO: 0051726	Regulation of cell cycle	KIF23, CCNB1, E2F1, CLSPN, CCNE1, E2F2, PTGS2, SFRP1, E2F7, EZH2, DACH1, AXIN2	12	0.001

GO: 0003006	Developmental process involved in reproduction	CCNB1, PLAG1, PTGS2, SFRP1, E2F7, FOXF2, HOXA10, HOXA9, CBX2, DACH1	10	0.001

GO: 0022414	Reproductive process	E2F1, CCNB1, PLAG1, PROK2, SFRP1, PTGS2, E2F7, FOXF2, HOXA10, HOXA9, CBX2, DACH1, ACSL4	13	0.002

GO: 0000003	Reproduction	E2F1, CCNB1, PLAG1, PROK2, SFRP1, PTGS2, E2F7, FOXF2, HOXA10, HOXA9, CBX2, DACH1, ACSL4	13	0.002

GO: 0010564	Regulation of cell cycle process	KIF23, CCNB1, E2F1, CLSPN, SFRP1, E2F7, EZH2, DACH1, AXIN2	9	0.010

GO: 0022402	Cell cycle process	KIF23, CCNB1, E2F1, CLSPN, CCNE1, E2F7, RRM2, EZH2, PBK, DACH1, CEP55, AXIN2	12	0.015

GO: 0051171	Regulation of nitrogen compound metabolic process	E2F1, PLAG1, E2F2, PTGS2, CPEB3, E2F7, EZH2, ELAVL2, CBX2, DACH1, CCNB1, CCNE1, HOXA3, SFRP1, RRM2, FOXF2, HOXA10, HOXA9, AXIN2, POLQ	20	0.020

GO: 1903047	Mitotic cell cycle process	KIF23, CCNB1, E2F1, CLSPN, CCNE1, E2F7, RRM2, EZH2, PBK, CEP55	10	0.022

GO: 0005667	Transcription factor complex	E2F1, E2F2, E2F7, FOXF2, HOXA10, HOXA9, DACH1	7	0.023

Note: FDR < 0.05. GO: Gene Ontology; DEmRNAs: differentially expressed mRNA; ceRNA: competing endogenous RNA; DAVID: Database for Annotation, Visualization, and Integrated Discovery; FDR: False discovery rates.

**Table 3:** KEGG pathway analysis for DEmRNAs involved in the ceRNA network using the ClusterProfiler package of R.

KEGG ID	Pathway name	Genes	Gene count	FDR
hsa04110	Cell cycle	CCNB1, CCNE1, E2F1, E2F2	4	0.004
hsa04218	Cellular senescence	CCNB1, CCNE1, E2F1, E2F2	4	0.005
hsa04115	p53 signaling pathway	CCNB1, CCNE1, RRM2	3	0.005
hsa04114	Oocyte meiosis	CCNB1, CCNE1, cpeb3	3	0.019
hsa04216	Ferroptosis	ACSL4, SLC7A11	2	0.020
hsa00480	Glutathione metabolism	G6PD, RRM2	2	0.032

Note: FDR < 0.05. KEGG: Kyoto Encyclopedia of Genes and Genomes.

**Table 4:** The list of DEmRNAs and DElncRNAs involved in the ceRNA network associated with overall survival in patients with stage I HCC.

Names	Genes	Regulation	logFC
CEP55	mRNA	Up	2.567
E2F1	mRNA	Up	3.502
E2F7	mRNA	Up	3.124
EZH2	mRNA	Up	2.240
G6PD	mRNA	Up	2.180
SLC7A11	mRNA	Up	4.165
AC061975.6	lncRNA	Up	3.587
AC079341.1	lncRNA	Up	3.097
CLLU1	lncRNA	Up	3.610
BPESC1	lncRNA	Up	2.876

## Data Availability

The data used to support the findings of this study are included within the article.
